# Telehealth vs In-Person Outpatient Mental Health Service Use and Spending Among Medicare Beneficiaries From 2019 to 2023

**DOI:** 10.1001/jamanetworkopen.2025.52239

**Published:** 2026-01-05

**Authors:** Ryan K. McBain, Jonathan Cantor, Rose Kerber, Cheryl L. Damberg, Christopher M. Whaley

**Affiliations:** 1 Healthcare Division, RAND, Arlington, Virginia; 2 Healthcare Division, RAND, Santa Monica, California; 3 Healthcare Division, RAND, Providence, Rhode Island; 4 Department of Health Services, Policy, and Practice, Brown School of Public Health, Providence, Rhode Island

## Abstract

This cross-sectional study identifies patterns of telehealth utilization and spending for anxiety, depression, bipolar disorder, schizophrenia, and posttraumatic stress disorder before, during, and after the COVID-19 pandemic among Medicare fee-for-service beneficiaries.

## Introduction

During the COVID-19 pandemic, the Centers for Medicare & Medicaid Services relaxed restrictions on telehealth services, leading to rapid expansion of telehealth use for mental health care.^1^ Although postpandemic patterns are documented for commercially insured populations,^2^ little is known about Medicare fee-for-service (FFS) beneficiaries,^3^ who represent a substantial share of mental health care users. To fill the gap, we examined monthly telehealth and in-person outpatient mental health service utilization and spending rates among FFS beneficiaries. We focused on 5 major diagnostic categories representing approximately 75% of assigned diagnoses for individuals meeting criteria for any mental disorder.^4^

## Methods

We conducted a retrospective analysis of all Medicare FFS beneficiaries with any mental health–related outpatient visits from any clinician between January 1, 2019, and December 31, 2023. Data were drawn from FFS carrier claims and restricted by using outpatient and telehealth place-of-service codes 02, 10, 11, 22, 23. The Brown University IRB deemed this cross-sectional study exempt from review and waived informed consent because deidentified, secondary data were used. We followed the STROBE reporting guideline.

Diagnoses were defined using *International Statistical Classification of Diseases and Related Health Problems, Tenth Revision* codes for anxiety disorders (F41), depressive disorders (F32, F33), bipolar spectrum disorders (F31), schizophrenia spectrum disorders (F25), and posttraumatic stress disorder (F43). Claims were included if a relevant diagnosis appeared as the primary or secondary; when both diagnoses were eligible, the primary was assigned. Data were segmented into (COVID-19) prepandemic (January 2019-February 2020), acute pandemic (March-December 2020; before vaccine access), and postpandemic (January 2021-December 2023) periods.

We estimated condition-specific trends using segmented ordinary least squares linear regression. Primary specification regressed outcomes (monthly claims rates per 100 000 beneficiaries; monthly spend rates [2023 dollars] per 10 000 beneficiaries) on indicators for pandemic and postpandemic periods, with intercepts representing prepandemic monthly rates. To accommodate serial correlation, we used Newey-West SEs with a 12-month bandwidth. In the sensitivity analysis, we adjusted for seasonality using month-of-year fixed effects.

Two-sided *P* < .05 indicated statistical significance. Analyses were completed in Stata 18 (StataCorp).

## Results

We included 9 548 926 beneficiaries (6 388 231 females [66.9%]), representing $8.1 billion in spending. Before the pandemic, telehealth comprised 2.1% of outpatient mental health claims. This rate increased to 54.4% (*P* < .001) during the pandemic then declined to 42.9% (*P* < .001) after the pandemic. The pattern was consistent across diagnostic categories (eg, for depression, telehealth represented 2.4% of claims before, 57.3% during, and 44.2% after the pandemic) (Table). Modeled estimates showed total mental health claims remained stable over time, averaging 9033 claims per 100 000 beneficiaries per month during the prepandemic, 10 642 during the pandemic, and 9395 during the postpandemic periods (Figure).

**Table.** Changes in Telehealth and In-Person Mental Health Service Claims and Spend Rates Before, During, and After the COVID-19 Pandemic, 2019-2023

Diagnostic category	Prepandemic period^a^		Pandemic period^b^		Postpandemic period^b^	
	β-Coefficient (95% CI)	*P* value	β-Coefficient (95% CI)	*P* value	β-Coefficient (95% CI)	*P* value
**Monthly claims per 100 000 beneficiaries, No.**						
Depression						
In-person	3354.5 (3488.4 to 3580.6)	<.001	−1672.6 (−1848.4 to −1496.9)	<.001	241.7 (65.4 to 417.9)	.008
Telehealth	81.5 (79.2 to 83.7)	<.001	2179.1 (2005.4 to 2352.7)	<.001	−734.7 (−997.3 to −472.1)	<.001
Anxiety						
In-person	2361.1 (2343.5 to 2378.8)	<.001	−824.1 (−887.4 to −760.8)	<.001	205.1 (98.9 to 311.4)	<.001
Telehealth	31.6 (29.5 to 33.8)	<.001	1369.2 (1205.0 to 1533.5)	<.001	−439.4 (−630.0 to −248.8)	<.001
Bipolar disorder						
In-person	1174.5 (1138.0 to 1211.0)	<.001	−650.2 (−751.9 to −548.6)	<.001	−1.2 (−75.4 to 73.0)	.97
Telehealth	36.7 (36.2 to 37.2)	<.001	839.0 (779.2 to 898.8)	<.001	−309.0 (−423.6 to −194.4)	<.001
Schizophrenia						
In person	511.2 (500.4 to 521.9)	<.001	−254.3 (−294.1 to −214.5)	<.001	16.4 (−14.6 to 47.4)	.30
Telehealth	25.3 (25.1 to 25.6)	<.001	329.7 (305.3 to 354.0)	<.001	−138.3 (−188.7 to −88.0)	<.001
PTSD						
In person	1257.9 (1243.1 to 1272.7)	<.001	−674.4 (−740.3 to −608.5)	<.001	135.6 (42.8 to 228.4)	.005
Telehealth	18.4 (16.5 to 20.3)	<.001	967.8 (911.9 to 1023.6)	<.001	−222.5 (−296.5 to −148.6)	<.001
Total						
In person	8839.2 (8736.7 to 8941.7)	<.001	−4075.7 (−4511.7 to −3639.6)	<.001	597.6 (155.3 to 1039.8)	.009
Telehealth	193.5 (186.8 to 200.3)	<.001	5684.7 (5220.2 to 6149.2)	<.001	−1844.0 (−2515.8 to −1172.2)	<.001
**Monthly spending per 10 000 beneficiaries, $**						
Depression						
In person	28 777.5 (27 038.0 to 30 517.1)	<.001	1252.1 (−485.6 to 2989.9)	.16	331.5 (−826.5 to 1489.6)	.57
Telehealth	424.4 (395.7 to 453.0)	<.001	5692.2 (5126.9 to 6257.5)	<.001	−1462.4 (−2055.7 to −869.1)	<.001
Anxiety						
In person	18 265.2 (16 891.4 to 19 369.0)	<.001	3022.4 (1428.7 to 4616.1)	<.001	805.2 (−511.9 to 2122.3)	.23
Telehealth	179.2 (167.8 to 190.7)	<.001	2909.5 (2621.7 to 3197.2)	<.001	−364.8 (−697.2 to −32.4)	.03
Bipolar disorder						
In person	8421.1 (7858.8 to 8983.3)	<.001	64.3 (−510.9 to 639.6)	.83	−993.6 (−1534.6 to −452.7)	<.001
Telehealth	161.6 (149.2 to 174.0)	<.001	2108.8 (1908.7 to 2309.0)	<.001	−646.5 (−875.5 to −417.5)	<.001
Schizophrenia						
In person	3179.5 (2950.3 to 3408.7)	<.001	−11.3 (−238.7 to −216.2)	<.001	−264.6 (−423.6 to −105.6)	.002
Telehealth	109.5 (100.2 to 118.8)	<.001	801.0 (730.5 to 871.5)	<.001	−286.4 (−375.5 to −197.4)	<.001
PTSD						
In-person	11 493.5 (10 772.8 to 12 214.1)	<.001	865.9 (175.0 to 1566.9)	.02	168.5 (−334.8 to 671.7)	.51
Telehealth	97.8 (85.1 to 110.6)	<.001	3188.5 (2948.0 to 3428.9)	<.001	−497.4 (−769.0 to −225.7)	<.001
Total						
In person	70 136.8 (65 588.7 to 74 684.9)	<.001	5193.5 (607.8 to 9779.3)	.03	47.0 (−2788.7 to 2882.7)	.97
Telehealth	972.5 (901.6 to 1043.5)	<.001	14 700.0 (13 343.4 to 16 056.6)	<.001	−3257.5 (−4670.7 to −1844.2)	<.001

Abbreviation: PTSD, posttraumatic stress disorder.

^a^ Coefficients reported for the prepandemic period correspond to intercept terms in regression models: mean monthly claims (and spend) rate during this interval. ^b^Coefficients reported during the pandemic and postpandemic periods are additional intercept terms in regression models, indicating the magnitude of change in mean monthly claims (and spend) rate during these intervals, compared with the prepandemic period.

**Figure.**
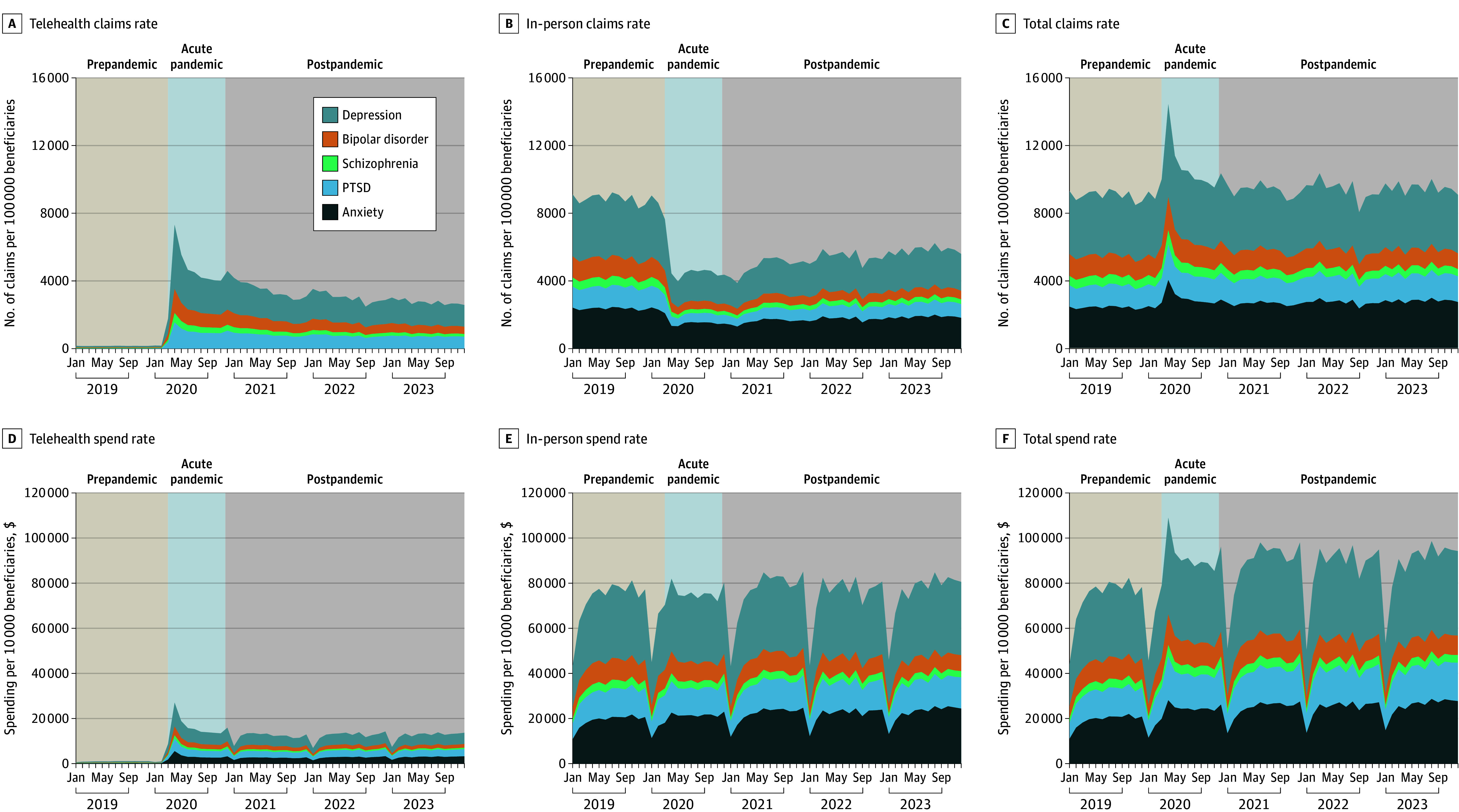
Telehealth and In-Person Outpatient Mental Health Service Claims and Spend Rates Among Medicare Fee-for-Service Beneficiaries From 2019 to 2023 Pre–(COVID-19) pandemic period extended from January 2019 to February 2020; acute pandemic extended from March to December 2020, and postpandemic period extended from January 2021 to December 2023. PTSD indicates posttraumatic stress disorder.

Estimates indicated outpatient mental health spending increased during the pandemic from $71 109 to $91 003 per 10 000 beneficiaries per month (*P* < .001). Spending remained elevated ($87 792) in the postpandemic period. Overall, telehealth mental health spending increased during vs before the pandemic—from $973 to $15 673 per 10 000 beneficiaries per month—and stabilized at $12 415 after the pandemic.

## Discussion

This study characterized trends in outpatient mental health care among FFS beneficiaries over a 5-year period of unprecedented health care disruptions. Before the pandemic, only 2.1% of services were delivered via telehealth. After the pandemic, this share was 42.9%. Nevertheless, total service utilization remained stable, suggesting telehealth visits have been largely substitutionary.^5^ Meanwhile, spending increased by 11%. Alongside prior studies,^2,6^ these findings indicate mental health telehealth has become a durable care component among beneficiaries.

Study limitations include, first, a Medicare FFS focus. Medicare Advantage enrollees differ in demographic and clinical backgrounds and may exhibit divergent service patterns. Second, we did not assess clinical outcomes or disaggregate findings by older and younger cohorts. Third, alternatively defined time frames for pandemic analysis could alter reported estimates. Lastly, study inclusion depended on recorded outpatient service and primary or secondary diagnosis in carrier claims.

## Notes

Supplement.
Data Sharing Statement
