# Supplementary material for: Telehealth vs In-Person Outpatient Mental Health Service Use and Spending Among Medicare Beneficiaries From 2019 to 2023
Source: JAMA Netw Open. 2026 Jan 5;9(1):e2552239. doi: 10.1001/jamanetworkopen.2025.52239 (PMC12771218; doi:10.1001/jamanetworkopen.2025.52239)
Supplement: Supplement. — Data Sharing Statement [file jamanetwopen-e2552239-s001.pdf]

Data Sharing Statement

McBain. Telehealth vs In-Person Outpatient Mental Health Service Use and Spending Among Medicare Beneficiaries From 2019 to 2023. *JAMA Netw Open*. Published January 05, 2026. doi:10.1001/jamanetworkopen.2025.52239

Data
Data available: No

Additional Information
Explanation for why data not available: These data derived from CMS and would require individuals to request restricted access data from CMS.
